# Endothelial Induced EMT in Breast Epithelial Cells with Stem Cell Properties

**DOI:** 10.1371/journal.pone.0023833

**Published:** 2011-09-06

**Authors:** Valgardur Sigurdsson, Bylgja Hilmarsdottir, Hekla Sigmundsdottir, Agla J. R. Fridriksdottir, Markus Ringnér, Rene Villadsen, Ake Borg, Bjarni A. Agnarsson, Ole William Petersen, Magnus K. Magnusson, Thorarinn Gudjonsson

**Affiliations:** 1 Stem Cell Research Unit, Biomedical Center, School of Health Sciences, University of Iceland, Reykjavik, Iceland; 2 Department of Laboratory Hematology, Landspitali University Hospital, Reykjavik, Iceland; 3 Department of Cellular and Molecular Medicine, Centre for Cell Biological Disease Analysis, and the Danish Stem Cell Centre, Faculty of Health Sciences, University of Copenhagen, Copenhagen, Denmark; 4 Department of Oncology, Clinical Sciences, Lund University, Lund, Sweden; 5 Department of Pathology, Landspitali University Hospital and School of Health Sciences, University of Iceland, Reykjavik, Iceland; Garvan Institute of Medical Research, Australia

## Abstract

Epithelial to mesenchymal transition (EMT) is a critical event in cancer progression and is closely linked to the breast epithelial cancer stem cell phenotype. Given the close interaction between the vascular endothelium and cancer cells, especially at the invasive front, we asked whether endothelial cells might play a role in EMT. Using a 3D culture model we demonstrate that endothelial cells are potent inducers of EMT in D492 an immortalized breast epithelial cell line with stem cell properties. Endothelial induced mesenchymal-like cells (D492M) derived from D492, show reduced expression of keratins, a switch from E-Cadherin (E-Cad) to N-Cadherin (N-Cad) and enhanced migration. Acquisition of cancer stem cell associated characteristics like increased CD44^high^/CD24^low^ ratio, resistance to apoptosis and anchorage independent growth was also seen in D492M cells. Endothelial induced EMT in D492 was partially blocked by inhibition of HGF signaling. Basal-like breast cancer, a vascular rich cancer with stem cell properties and adverse prognosis has been linked with EMT. We immunostained several basal-like breast cancer samples for endothelial and EMT markers. Cancer cells close to the vascular rich areas show no or decreased expression of E-Cad and increased N-Cad expression suggesting EMT. Collectively, we have shown in a 3D culture model that endothelial cells are potent inducers of EMT in breast epithelial cells with stem cell properties. Furthermore, we demonstrate that basal-like breast cancer contains cells with an EMT phenotype, most prominently close to vascular rich areas of these tumors. We conclude that endothelial cells are potent inducers of EMT and may play a role in progression of basal-like breast cancer.

## Introduction

Epithelial to mesenchymal transition (EMT) is associated with increased aggressiveness and adverse prognosis in carcinomas [1], [2]. This conversion of cancer cells towards a more mesenchymal phenotype involves loss or lowered expression of epithelial markers (e.g. E-Cad and keratins), increased expression of mesenchymal markers (e.g. N-Cad, vimentin, fibronectin), increased mobility and an invasive phenotype [3], [4], [5]. EMT in breast cancer is tightly linked to the triple negative (ER-, PR- and ErbB2-) basal-like breast cancer subgroup and cancer stem cells [6], [7], [8], [9], [10], [11], [12]. Basal-like breast cancers express many markers associated with both myoepithelial and luminal epithelial cells suggesting the bipotential differentiation pattern and possible stem cell origin of these tumors [9], [13], [14]. Previous studies have demonstrated increased expression of EMT markers at tumor-stroma interfaces [15], [16] and stromal cells are increasingly being recognized as major players in cancer progression [17], [18]. Increasing number of factors are known that can induce EMT including transforming growth factor-β (TGF-β), ligands for receptor tyrosine kinases such as vascular endothelial growth factor (VEGF), epidermal growth factor (EGF) and hepatocyte growth factor (HGF) as well as components of the extracellular matrix [3], [19]. These signaling events ultimately control transcriptional regulatory factors such as Snail, Slug, Twist, ZEB1, ZEB2 and FOXC2 leading to increased and decreased expression of mesenchymal and epithelial markers, respectively. Defining the cellular and microenvironmental cues that trigger EMT during the progression of breast cancers is critical and could provide new therapeutic targets.

Vascular endothelial cells have attracted increased attention as important regulators of organogenesis and stem cell maintenance in various tissues, such as bone marrow, brain, liver and pancreas [20], [21], [22], [23]. Furthermore, intratumoral angiogenesis is also one of the hallmarks of cancer progression and increased microvessel density in tumors is an indicator of poor prognosis [12]. In the breast gland, Shekhar et al. have previously shown that human umbilical vein endothelial cells (HUVEC) induce ductal-alveolar morphogenesis of preneoplastic MCF10A cells [24]. We have recently improved methods to propagate breast endothelial cells (BRENCS) in culture and shown that BRENCS can mediate proliferative and morphogenic signals to breast epithelial cells in coculture [25], [26]. In the lung, we have shown that endothelial cells induce branching morphogenesis in lung epithelial cells when cocultured in a 3D model. Interestingly, these structures mimic phenotypic traits of lung histology *in vivo* including bronchio-alveolar like structures [27]. Thus, data from diverse organs shows that endothelial cells are important players in tissue remodeling making this cell type particularly interesting as a regulator of morphogenesis.

We have previously established a breast epithelial cell line, referred to as D492, which has a basal-like phenotype as evidenced by expression of both luminal (K8, K19) and myoepithelial (K5/6, K14) cytokeratins. Furthermore, D492 has stem cell properties as demonstrated by its ability to differentiate into luminal- and myoepithelial cells and to form branching TDLU-like structures in a 3D reconstituted basement membrane (rBM) [28], [29]. Here, we demonstrate in 3D coculture that endothelial cells are potent inducers of EMT in D492 and this process is partially inhibited by blocking HGF. Furthermore, we show in basal-like breast cancer that N-Cad a marker of EMT is upregulated in proximity to vascular rich areas. These data suggest that the vascular rich stroma in breast cancer lesions might serve as an ideal niche for the stimulation of epithelial cancer cells to undergo EMT, and might especially apply to the highly aggressive basal-like breast cancers, a subtype rich in stem cells.

## Materials and Methods

### Cell culture

D492 and D382 were cultured in H14 medium as described previously [28]. W2320 cell line was cultured in DMEM/F12+5% FBS [33]. The MCF-7, MCF10A and MDA-MB-231 cell lines where purchased from ATCC (American Type Culture Collection) and are routinely authenticated with genotype profiling according to ATCC guidelines. Primary human BRENCs were isolated from breast reduction mammoplasties as previously described by Sigurdsson et al. [25] and cultured on endothelial growth medium (EGM) (Lonza) containing 50 IU/ml penicillin, 50 µg/ml streptomycin, hydrocortisone, FGF, EGF, VEGF, R3-IGF-1, Ascorbic acid, Heparin, GA-1000 and supplemented with 5% FBS (EGM5). Growth factor reduced reconstituted basement membrane (rBM, purchased as Matrigel, BD Biosciences) was used in direct 3D coculture. Transwell coculture was conducted in a 24 well setup with a 0,4 µm polyester membrane seperating the chambers (Costar). 5×10^4^ endothelial cells were seeded in the upper chamber as a monolayer and 250 D492 cells in 100 µl matrigel on the bottom of the lower chamber maintained on EGM5. For additional information on cell culture and 3D coculture see Methods S1.

### Blocking experiments

Direct coculture of 500 D492 cells with 2×10^5^ BRENCs in 300 µl of rBM were treated with 8 µg/ml anti-HGF neutralizing antibody (#MAB294, R&D Systems) in the rBM and in the medium. In transwell coculture HGF was blocked with 8 µg/ml anti-HGF in the rBM and in the medium in the lower transwell chamber and the controls were treated with mouse IgG1 in the same manner.

### Immunochemistry and tumor samples

Formalin-fixed, paraffin embedded tissue blocks were cut into 5 µm serial sections and mounted on slides. Sections were deparaffinized and rehydrated in xylene and ethanol. Antigen retrieval was done by boiling in citrate buffer for 15 min. The following primary antibodies were used; fibronectin (LabMab, gift from D.E. Mosher [30], CD-31 (M0823, DakoCytomation), Keratin 19 (ab7754, Abcam), Keratin 14 (NCL-LL002, NovoCastra), E-Cad (#13-1700, Zymed), N-Cad (#610920, BD), EpCAM (NCL-ESA, Novocastra). For double and triple labelling experiments we used fluorescence iso-type specific secondary antibodies (Invitrogen). Fluorescent nuclear counterstain, TO-PRO-3 (Invitrogen) was used in immunofluorescence. Specimens were visualized on a Zeiss LSM 5 Pascal laser-scanning microscope (Carl Zeiss). Breast cancer specimens were from the clinical Department of Pathology, Landspitali, University Hospital and included 9 basal-like and four estrogen receptor positive (ER-positive) breast cancers. This work has been approved by the National Bioethics Committee of Iceland, Reference number VSNa2001050056.

### Western blotting

Equal amounts (5 µg) of proteins were separated on 10% NuPage Bis-Tris gels (Invitrogen) and transferred to a PVDF membrane (Invitrogen). Antibodies: E-Cad (1∶500; Zymed), N-Cad (1∶1000; BD), β-actin (1∶5000; Abcam), GAPDH (1∶5000; Abcam), K5/6 (1∶1000; Zymed), K8 (1∶1000; Abcam), K14 (1∶1000; Abcam), α-SM-Actin (1∶500; Dako) K17 (1∶500; Dako), K19 (1∶1000; abcam), Vimentin (1∶1000;Dako) and FOXC2 (1∶2000; Abcam) were used. Membranes were visualized with ECL+ after incubation with anti-mouse or rabbit secondary antibody (1∶5000) (GE healthcare).

### Migration, anchorage independence and mammosphere assays

For migration experiments a total of 1×10^4^ and 2,5×10^4^ starved cells were seeded in DMEM/F12 basic medium on collagen coated transwell filter in a transwell Boyden chamber (Corning) with an 8 µm pore size. The transwell filter were incubated in collagen (0.06 µg/µl) in PBS for 24 h at 4°C, then excess collagen solution was rinsed off with PBS before cells were seeded. EGM5 medium was used as a chemoattractant in the lower chamber. After 12 h incubation cells in the upper chamber were removed with a cotton swab and migrated cells on the bottom surface stained with 0.1% crystal violet. Cells were counted in three representative fields in each transwell. Soft agar assay was performed by mixing 1×10^4^ D492 and D492M cells to 1.5 ml of 0.5% low melting agar (Invitrogen) that was overlaid on 1% agar solution in 6 well plates and cultured on H14 medium. After 20 days the colonies were stained with crystal violet and counted. Mammosphere assay was done in 24 well Ultra-Low attachment plates (Corning) where 500, single cell filtered, D492 and D492M cells were seeded and cultured on EGM5 medium. Number and size of spheres was evaluated after 8 days.

### Apoptosis resistance

D492 and D492M were seeded into 6 well culture plates (BD) and grown to 70% confluency. Cells were treated with 10 µM of Camptothecin (Sigma) in EGM5 medium and counted on culture days 0–3.

### Flow cytometry analysis

Adherent cells were trypsinized and filtered through a 30 nm nylon filter (Millipore). Cells were incubated for 20 minutes with fluorochrome-conjugated antibodies against CD44 (clone IM7, BD), CD24 (clone ML5, BD) or isotype-matched controls, subsequently washed and resuspended in PBS with 4% formaldehyde (cell-fix). Cells were collected (2×10^4^ events) on a FACS-Calibur (BD) and analysed using CellQuest (BD).

### Statistical analysis

Data is presented as mean +SEM from number of independent experiments as indicated. Statistical analysis was performed by two-tailed Students T-test using GraphPad. P values of <0.05 were considered to be statistically significant.

## Results

### Immortalized breast epithelial cell line with stem cell properties generate mesenchymal-like cells in coculture with endothelial cells

**Figure 1:**
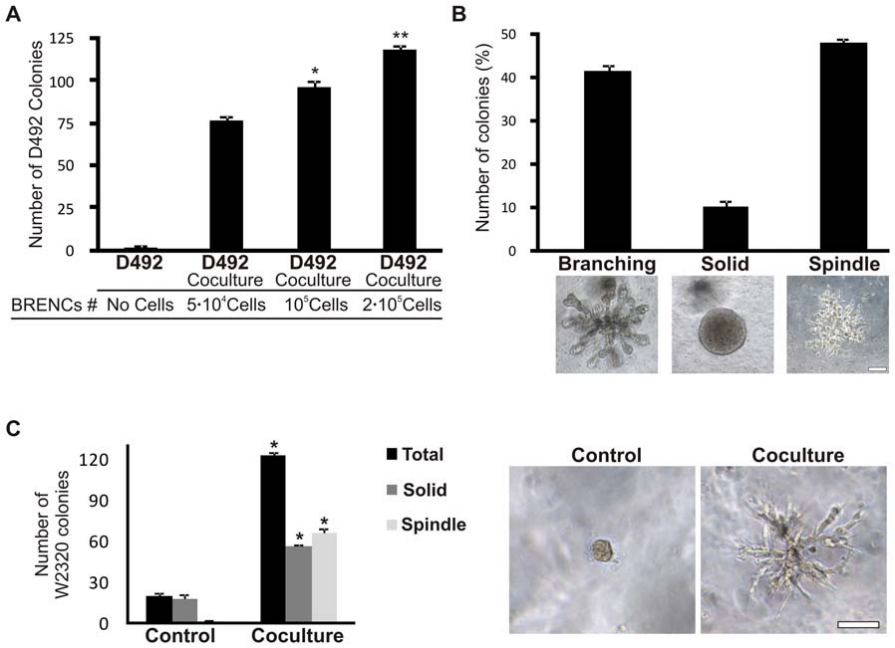
Breast epithelial cells with stem cell properties generate spindle-like cells in coculture with endothelial cells. **A**, Colony growth of D492-derived epithelial structures increases proportional with increased number of BRENCs in coculture. When 500 D492 cells are cultured in 300 µl rBM they fail to grow (control). With BRENCs, colony growth increases from 76 (5×10^4^ BRENCs), 96 (1×10^5^ BRENCs) to 117 colonies (23,5% cloning efficacy) when 2×10^5^ BRENCs are inoculated with 500 D492 cells. Average (AVG) number of colonies +SEM in three experiments. *, p<0.05; **, p<0.01; compared to 5×10^4^ BRENCs. **B**, D492 generate spindle-like cells in coculture with BRENCs (2×10^5^ cells). D492 cells (500 cells incubated) form three distinct structures, branching, solid, and spindle-like colonies. Appearance of the spindle colonies from D492 is novel and occurs only in coculture with endothelial cells. Average % of colony type +SEM in three experiments. Bar 100 µm. **C**, Using a primary metaplastic breast cancer cell line, W2320, we were able to show that these cells could also produce spindle-like colonies in coculture with BRENCs (right). Data shown as AVG number of colonies +SEM in three experiments (left). *p<0.05. Bar 100 µm.

The D492 cell line forms branching structures in reconstituted basement membrane (rBM) [28], [29]. Growth of D492 alone in rBM requires, however, moderate cell density (1×10^4^ cells per 300 µl rBM) [28]. In order to test the effects of breast endothelial cells (BRENCs) on growth, and morphogenesis of D492 cells we set up a coculture with BRENCS and D492 cells inside a rBM. In this assay BRENCs remain viable and metabolically active but non-proliferative (Fig S1). No growth was seen when D492 cells were cultured alone at clonal dilution (500 cells per 300 µl rBM) (Fig. 1A). In contrast, in coculture with BRENCs the total number of D492 colonies increased with increasing amount of endothelial cells reaching a cloning efficacy of 23.5% (117.3±3.5 colonies; p<0.01) (Fig. 1A). In addition to solid round and branching structures that have previously been shown to form when D492 are cultured alone, spindle shape, mesenchymal-like colonies emerged in coculture with BRENCs (Figs. 1B and S2). No effect was seen on endothelial cell morphology under coculture conditions. These data suggest that BRENCs stimulate growth and morphogenesis of D492 and furthermore induce the formation of spindle-shaped colonies reminiscent of EMT in a 3D environment.

To see if the endothelial induced EMT-like phenotype was breast-endothelial specific we also cocultured D492 with human umbilical vein endothelial cells (HUVECs). HUVECs were also able to induce a similar phenotype to what was seen in coculture with BRENCs (data not shown) suggesting a general endothelial-derived effect rather than an endothelial organ-specific effect.

As D492 has an immunophenotype similar to the cells of basal-like breast cancer, we also tested W2320 which is a basal-like metaplastic breast cancer cell line [31]. W2320 generated solid epithelial colonies when cultured alone in 3D rBM. In contrast, when cocultured with BRENCs there was a marked increase in total colony formation and induction of spindle-like colonies (Fig. 1C). We also tested several other cell lines in our 3D coculture model. D382 is E6E7 immortalized cell line generated from differentiated, normal, luminal breast epithelial cells [28] and MCF10A is a non-tumorigenic epithelial cell line. MCF-7, is an estrogen receptor positive breast cancer cell line, while MDA-MB-231 is a highly malignant basal-like breast cancer cell line. When these cell lines were cocultured with BRENCs in a rBM assay, MDA-MB-231 generated mesenchymal colonies while D382 and MCF10A, generated only round epithelial colonies (Fig. S3). Furthermore, the estrogen receptor positive breast cancer cell line MCF-7, generated only large solid round colonies in coculture with BRENCs (Fig. S3). This indicates that breast cancer cell lines with basal-like characteristics have the plasticity for mesenchymal conversion, in coculture with endothelial cells, while other more differentiated cell lines are unable to undergo this transition.

### Isolation and characterization of a D492-derived EMT cell line

**Figure 2:**
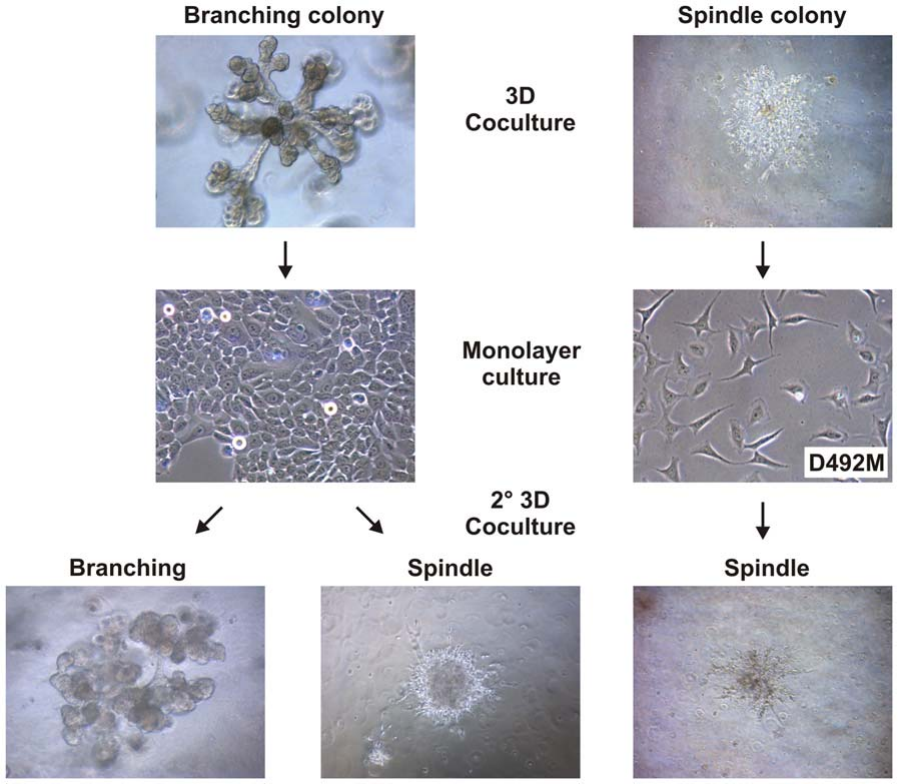
Isolation of D492-derived mesenchymal-like cells (D492M). Six branching and six spindle-like colonies were isolated and plated in monolayer culture. Cells from branching structures retain cuboidal epithelial phenotype in monolayer (left panel). When cocultured with BRENCs these cells generate branching TDLU-like (40%) and spindle-like colonies (50%) in secondary 3D culture (°2 3D). Cells from spindle-like colonies (right panel) showed mesenchymal/spindle like morphology in monolayer and cells isolated from one of these colonies gave rise to D492M. When cocultured with BRENCs these cells only gave rise to spindle like colonies in secondary 3D coculture.

To analyze the origin and morphogenic capacity of branching and spindle-like colonies from cocultures, we isolated single colonies and plated them into monolayer culture. Cells derived from branching colonies showed cuboidal epithelial phenotype whereas cells from spindle-like colonies showed a spindle shaped phenotype (Fig. 2). Spindle-like colonies were isolated and expanded as sublines, one of them is referred to as D492M (mesenchymal) (Fig. 2). When replated into secondary rBM cocultures, cells from spindle-like colonies were fixed in making similar colonies whereas cells from branching colonies retain the ability to make both branching and spindle-like colonies (Fig. 2).

The parental cell line D492 was initially established by transfection with a retroviral vector containing the E6 and E7 oncogenes and the neomycin resistant gene [28]. To eliminate possible endothelial-derived contamination, the D492M subline was selected in medium containing neomycin. Furthermore, we cloned and sequenced an insertion site of the retrovirus (Methods S1). We showed the presence of this insertion in D492M and four different single cell-derived mesenchymal colonies as well as being present in 5 different single cell derived D492 sub-clones (Fig. S4A). To further confirm the epithelial origin of the mesenchymal colonies we generated a D492 subline containing a GFP expressing vector. When these GFP positive D492 cells were cocultured with BRENCs all mesenchymal-like colonies were green (Fig. S4B). This confirms the epithelial origin of the mesenchymal colonies and furthermore confirms the clonal origin of D492M from the D492 cell line.

**Figure 3:**
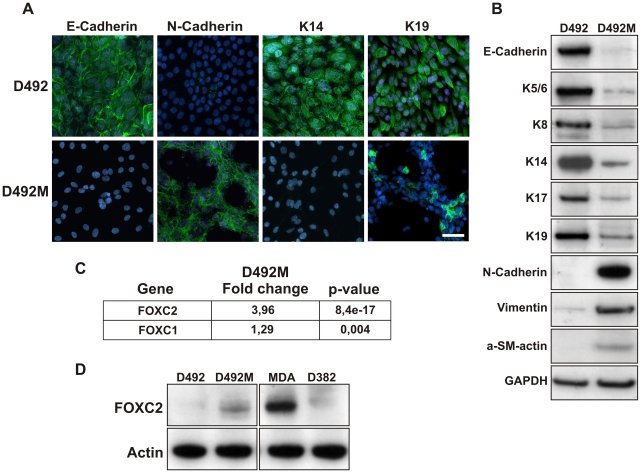
D492M has acquired an EMT phenotype. **A**, Immunofluoresence staining on D492M show switch from E- to N-Cad and reduced expression of K14 and K19. Counterstain TO-PRO-3, Bar 100 µm. **B**, Western blotting confirms downregulation of epithelial markers such as E-Cad, K-5/6, 8, 14, 17 and 19 in D492M. In contrast, the mesenchymal markers N-Cad, Vimentin and alpha-smooth muscle actin were expressed more intensively in D492M than D492. GAPDH loading control. **C**, EMT associated transcription factors are upregulated in D492M. Gene expression data showed upregulation of FOXC2 (3.96 fold,p: 8.4e-17) and FOXC1 (1.29 fold,p: 0.004) transcription factors in D492M. **D**, FOXC2 is strongly expressed in breast epithelial cell lines with EMT phenotype. Western blotting shows strong expression of FOXC2 in D492M and MDA-MB-231, an EMT-like breast cancer cell line, compared to no or low expression in D492 and D382. Actin, loading control.

Immunophenotypic characterization of D492M confirmed that the spindle cell morphology was a direct consequence of EMT. Thus, as opposed to the parent cell line, D492M has lost expression of E-Cad and shows reduced expression of keratins 5/6, 8, 14, 17, and 19, while showing increased expression of Vimentin, N-Cad, and alpha-smooth muscle actin (Figs. 3A and B). Using an *Illumina BeadChip* expression microarray (HumanWG-6 v3.0) we screened the expression pattern in the two cell lines. There was significantly different expression level of 9399 genes of the 13105 genes that had detectable expression levels (for an FDR of <1%). Clustering pattern for the top 50 genes demonstrates the clear differences between the two cell lines (Fig. S5). E-Cad, keratins 5, 6, 14, and 19 were all downregulated in D492M compared to D492. Likewise, mesenchymal markers such as N-Cad, Thy-1, thrombin receptor (PAR1), and CD70 were all highly up-regulated in D492M. Global gene expression shows EMT-associated transcription factors that are upregulated in D492M, including FOXC2 (3.96 fold), and FOXC1 (1.29 fold) (Fig. 3C). FOXC2 upregulation in D492M was confirmed with western blot and compared to D492, MDA-MB-231 and D382 (Fig. 3D). To confirm that the EMT is causally driven by the endothelial-induced EMT, rather than reflecting the properties of a single clonal cell sub-line we isolated four other sublines from D492 derived spindle-like colonies (D492M1-4). All these sublines were shown to have acquired an EMT phenotype (Fig. S6).

### D492M has acquired a functional EMT and cancer stem cell phenotype

**Figure 4:**
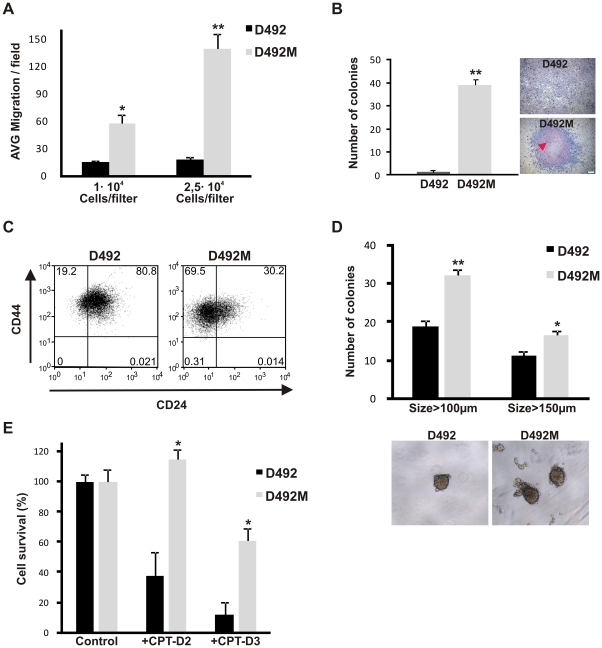
D492M has acquired cancer stem cell-like phenotype. **A**, D492M show increased migration compared to D492. Increased migration was seen at the two cell concentrations (1×10^4^ and 2.5×10^4^). **B**, D492M grow anchorage independently. In 0.5% soft agar D492 cells fail to form colonies. In contrast, the D492M cells are able to grow, indicating acquisition of anchorage independent growth. In monolayer culture (right) D492 cells are contact inhibited while D492M piles up in the culture flask indicating lack of contact inhibition (arrows). Counterstain hematoxylin. Bar 100 µm. **C**, D492M cells are CD44^high^,CD24^low^ consisting with the breast cancer stem cell phenotype. D492 contain a subpopulation (19%) of cells that are CD44^high^, CD24^low^. This population increases to 70% in the D492M cell line. **D**, D492 and D492M differ in their ability to form mammospheres. Both D492 and D492M can generate colonies in mammosphere assay, however, D492M generates more and larger (>100 µm: 1.7 fold; >150 µm:1.5 fold) mammospheres than D492 cells. **E**, D492M cells show delayed chemically induced apoptosis. D492 and D492M show distinct responses to Camptothecin, an apoptosis inducing agent. D492 cells underwent immediate apoptosis and showed cell survival under 40% on day 2 while having no effect on D492M. On day 3 D492M cells showed cell survival of 60% where only few D492 cells were left. Data shown as AVG number of cells per field (A,E) or AVG number of colonies (B,D) +SEM in three experiments. *p<0.05; **p<0.01.

A major characteristic of the mesenchymal phenotype is increased motility. In a transwell migration assay when compared to D492, the D492M cells showed increased migration, 3.8 fold (p<0.05) and 7.4 fold (p<0.01) when plated at 1×10^4^ or 2.5×10^4^ cells, respectively (Fig. 4A). Functionally, the D492M cells also showed signs of transformation by growth in soft agar assay. While D492 fail to grow, D492M grew well in this assay showing 6% cloning efficacy (p<0.01) (Fig. 4B). In addition, when cultured in monolayer, D492M formed multilayered ridges further indicating a loss of contact inhibition (Fig. 4B, right). The CD44^high^, CD24^low^ phenotype has been associated with cancer stem cell phenotype in the breast [32] and recently EMT-like traits have been added to this profile [6], [7]. Flow cytometry analysis showed that the D492 cells contain a mixture of CD44^high^,CD24^high^ cells (81%) and CD44^high^,CD24^low^ cells (19%). In contrast, D492M showed marked increase in the proportion of CD44^high^, CD24^low^ cells (70%) (Fig. 4C).

Papers have demonstrated a strong correlation between the EMT phenotype and the ability to form mammospheres, an assay that functionally tests for breast stem cell properties [6],[33]. When cultured in low attachment plates both D492 and D492M generated mammospheres demonstrating the self-renewal and cancer stem cell properties of these cell lines, respectively (Fig. 4D). However, D492M generated significantly larger and higher number of colonies (size>100 µm; p<0.01 and size>150 µm; p<0.05) in this assay (Fig. 4D). One of the hallmarks of cancer stem cells and EMT is the acquisition of apoptosis resistance [6], [34]. D492M showed increased resistance (p<0.05) to chemically induced apoptosis (Fig. 4E). Thus, D492M has acquired phenotypic and functional characteristics of EMT cells and cancer stem cells.

### Endothelial induced EMT in D492 is generated through soluble factors partially mediated by HGF

**Figure 5:**
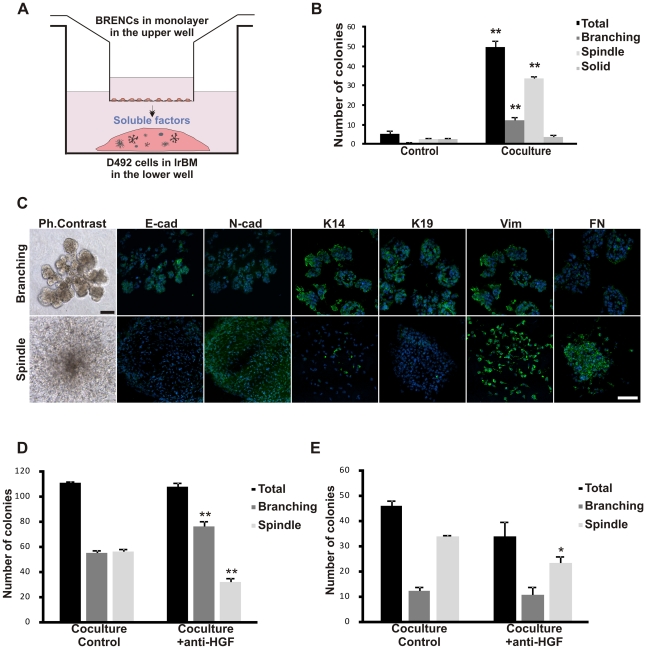
Endothelial induced EMT is mediated through soluble factors and is partially blocked by inhibition of HGF. **A**, In the transwell coculture setup endothelial cells were cultured as a monolayer in the upper well and D492 cells in 3D rBM on the bottom of the lower well. **B**, BRENCs induce spindle-like colony formation in transwell coculture. D492 cells without BRENCs showed limited growth (less than 1% of seeded cells, control). coculture the BRENCs induced a significant increase in number of spindle-like colonies. **C**, D492-derived branching colonies generated in transwell culture show characteristical epithelial phenotype including expression of E-Cad, K14 and K19. In contrast, D492-derived spindle like colonies show EMT phenotype including expression of N-Cad, Vimentin and fibronectin (FN). Bar 100 µm. **D–E**, Formation of spindle-like colonies is partially blocked by inhibition of HGF. Spindle-like colony formation is reduced with anti-HGF by 44% in direct coculture (D) and by 30% in transwell coculture (E). Data shown as AVG number of colonies +SEM in three experiments. *p<0.05; **p<0.01.

To analyze if endothelial induced EMT in D492 was mediated through soluble factors we used transwell coculture with BRENCs cultured on top of a filter and D492 cells embedded in rBM, in the lower well (Fig. 5A). In this setup, BRENCs were even more effective in inducing the emergence of spindle-like colonies (Fig. 5B) suggesting endothelial-derived soluble factor/s. These spindle-like colonies show an EMT phenotype with an E- to N-Cad switch, reduced K14 and K19 expression and increased expression of vimentin and fibronectin (Fig. 5C). It should, however, be noted that in this setup a few small colonies grew in D492 monoculture and were either of solid round or spindle-like morphology. The reason for this is unknown but may be due to the difference in the experimental setup of the transwell compared to the direct coculture 3D experiments.

There are a number of factors that can elicit EMT such as TGF-β1, FGF, EGF and HGF. As D492 did not form any EMT in the EGM5 coculture media that contains EGF, FGF and VEGF we set focused on TGF-β1 and HGF, known morphogenic and EMT inducing factors [3]. We treated 3D cocultures with a small molecule inhibitor targeting the TGF-β receptor-1 (ALK5) and with a TGF-β1 neutralizing antibody. We observed no changes in the number of spindle colonies using the ALK5 kinase inhibitor or the anti-TGF-β1 (not shown) indicating that other factors were responsible for the endothelial induced EMT.

HGF is expressed in endothelial cells and other stromal cells and can induce both scattering (including EMT) and morphogenic effects on epithelial cells [35]. In our 3D rBM assay BRENCs secreted HGF into the surrounding culture media as measured by ELISA. They secreted over four times higher concentrations than D492 in this setup (Fig. S7). When coculture of D492 and BRENCS was treated with a neutralizing antibody against HGF a significant decrease (p<0.01) in spindle colonies was observed in contrast to a significant increase (p<0.01) in the formation of branching colonies (Fig. 5D). We also tested this in transwell coculture and as before BRENCs induced the emergence of spindle colonies. Neutralizing antibody against HGF significantly decreased (p<0.05) their number but had no effects on branching colonies (Fig. 5E). Collectively, this suggests that the balance in formation of branching or spindle colonies from D492 cells can be modulated by HGF signaling and that soluble HGF, at least partially, mediates endothelial induced EMT in our 3D coculture model.

### EMT phenotype in basal-like breast cancers is associated with vascular-rich areas

**Figure 6:**
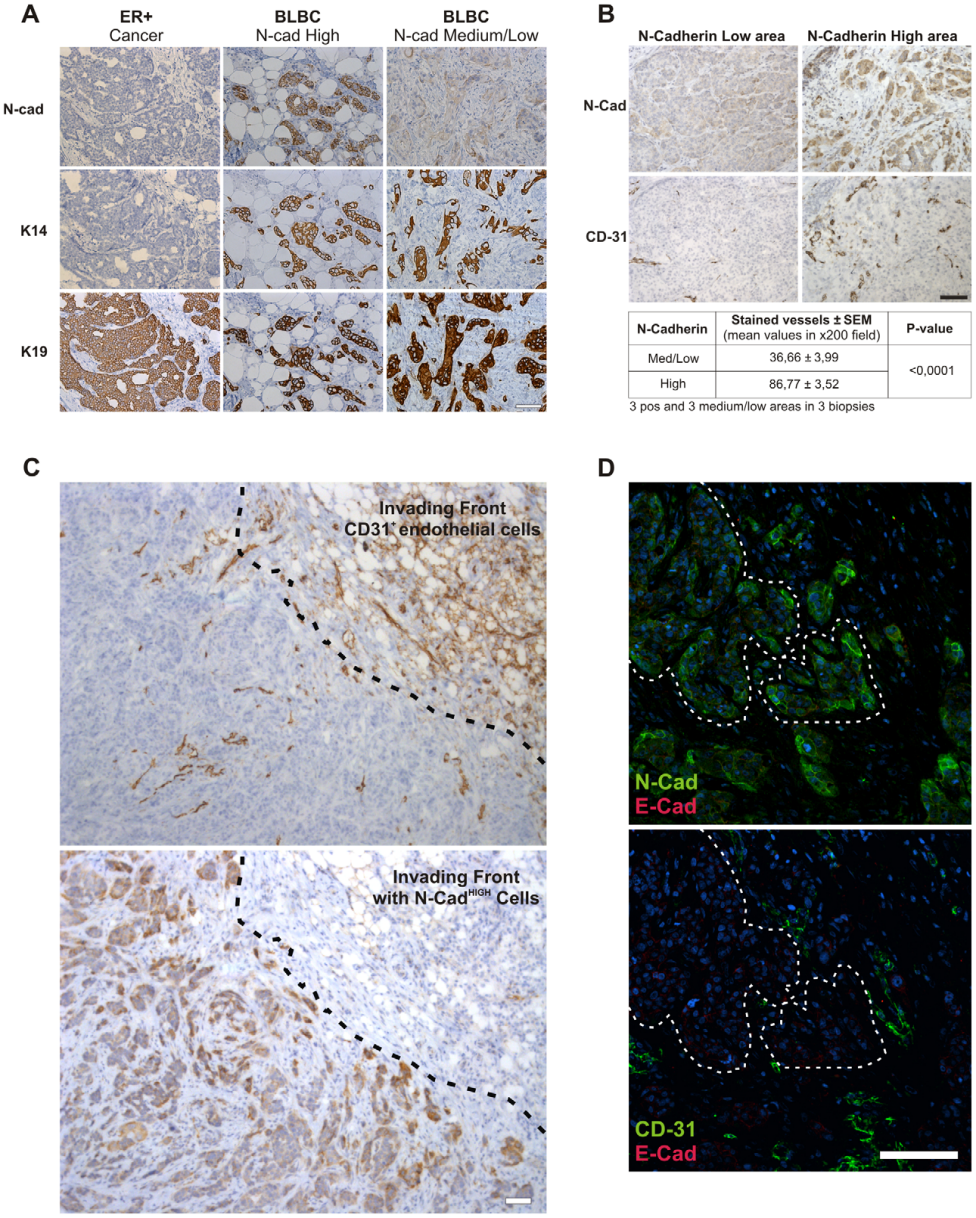
The EMT phenotype is most prominent close to vascular rich areas in basal-like breast cancer. **A**, N-Cad expression is most prominently found within basal-like breast cancer. ER tumors are K19 positive but negative for N-Cad and K14. In contrast basal-like breast cancers (BLBC) are positive for all three markers. Bar 100 µm. **B**, Increased microvessel density in basal-like breast cancer is associated with areas containing cells with high expression of N-Cad. Immunostaining show increased CD31 positive microvessels in areas with high N-Cad expression. Statistcal analyzis (bottom) from three basal-like breast cancer biopsies show significant increase in microvessels within areas with high N-Cad expression. **C**, Expression of CD-31 reveals highly vascularized area at the tumor stroma interface. N-Cad expression was seen in most cancer cells. Note the strong expression of N-Cad close to the vascular rich area (dashed line). **D**, Double-labeling against E- (red) and N-Cad (green) in the same area shows strong expression of N-Cad only. Low or no expression of E-Cad (red) was seen close to the CD31 positive (green) endothelial cells. Cells were counterstained with hematoxylin (A B and C) and TO-PRO-3 (D). Bar, 100 µm.

Circumstantial evidence suggests that basal-like breast cancers originate in epithelial stem or progenitor cells [14]. Furthermore, studies show that these tumors are highly vascularized [36], [37] and rich in EMT associated markers such as N-Cad with low or no E-Cad expression [9], [11]. Because both EMT and angiogenesis are associated with increased metastatic potential, we explored the possible connection between vascularization and the EMT phenotype within basal-like breast cancer. We stained 9 basal-like and four estrogen receptor positive (ER-positive) breast cancers with antibodies against E-Cad, N-Cad, K14, K19 and CD-31. While all ER-positive cancers were N-Cad and K14 negative, basal-like cancers were positive for N-Cad and K14, with some tumors showing medium-to-low expression of N-Cad (Fig. 6A). To study the possible association between vascularization and the EMT-phenotype, we quantified the microvascular density (MVD) in N-Cad medium-to-low areas and in N-Cad high areas. Microvessel density (MVD) was significantly higher in areas containing cells with high expression of N-Cad (MVD: 86.77±3.52) compared to areas with low N-Cad expression (MVD: 36.66±4.01) (Fig. 6B, 6C and Fig. S8). Low or no expression of E-Cad was seen in all basal-like biopsies tested (Fig. 6D). Thus the cellular context in basal like breast cancers reveals an interesting pattern of cancer cells showing an EMT phenotype closely associated with vascular rich components. Based on these findings we hypothesize that the endothelial compartment might contribute to the EMT phenotype of tumor cells within basal like breast cancer.

## Discussion

We report here, that in a 3D coculture model EMT-like cells arise from immortalized breast epithelial cells with stem cell properties upon interaction with breast endothelial cells. These effects are at least partially mediated through HGF with other endothelial-derived factors possibly involved. The endothelial induced transition resulted in a characteristic EMT phenotype as evidenced by marked difference in protein and gene expression with loss of many adhesion and epithelial specific markers and gain of mesenchymal markers. Functionally, the EMT cells showed increased migratory abilities and an increase in cancer stem cell phenotype. Furthermore, we show that basal-like breast cancers are rich in cells showing a potential EMT phenotype with highest intensity of N-Cad expression close to vascular rich areas.

EMT has recently been linked to basal-like breast cancer as demonstrated by upregulation of EMT markers (Vimentin, alpha-smooth muscle actin, and N-Cad) together with reduction of characteristic epithelial markers (E-Cad and keratins) [9], [11]. This is supported by our observation that basal like breast cancers have features of EMT as evidenced by no or reduced expression of E-Cad and high expression of N-Cad. Interestingly, the strongest expression of N-Cad was seen in vascular-rich areas suggesting that endothelial cells may provide a favorable environment for the EMT phenotype. Intratumoral angiogenesis, assessed by microvessel density, has been proposed to identify patients at high risk of recurrence, especially in node-negative breast cancer. Meta-analyses have confirmed this association, although being a relatively weak risk factor [38]. More recent studies have shown that microvessel density might be a major risk factor in triple negative breast cancer [39] and vascular endothelial growth factor (VEGF), a marker of angiogenesis, has also been shown to be significantly higher in this subclass of breast cancer [40]. High MVD has also been associated with medullary breast tumors, which are a subtype of the basal-like group and with breast tumors with a predominant CD44^high^/CD24^low^ cancer stem cell phenotype [37], [41]. Niu et al. have also showed in hepatocellular carcinoma, that tumors expressing Twist, a marker of EMT, have higher MVD [42].

EMT is a complex process and there have been numerous factors shown to elicit EMT in culture. Of these, TGF-β1 and ligands for various receptor tyrosine kinases have received much attention [34]. We report here that inhibition of TGF-β1 with a neutralizing antibody or an ALK5 inhibitor did not affect the formation of spindle-like colonies in coculture suggesting that TGF-β1 is not involved in endothelial induced EMT in the 3D-context. Interestingly, Mostov et al. reported that HGF induces partial EMT in MDCK cells cultured in 3D collagen gel [35]. The HGF receptor, c-Met has also been shown to have a higher expression in basal-like breast cancer than in other subtypes. Basal-like breast cancer are also enriched for gene sets indicating transcriptional activation induced by c-Met signaling [43]. Hypoxia, a major effector of endothelial cells has been shown to increase HGF mRNA stability through overexpression of HIF-1alpha [44]. Hypoxia has also been shown to increase the expression of c-Met, leading to increased sensitivity to HGF and an invasive phenotype in the tumor cells [45]. In our study, endothelial cells were shown to secrete HGF in 3D culture and when HGF was blocked with a neutralizing antibody in direct- and indirect (transwell) coculture a significant reduction in the number of EMT colonies was observed demonstrating that endothelial-derived HGF is, at least partially, responsible for EMT in our culture model. These findings suggest a novel role for endothelial cells and angiogenesis in cancer progression in addition to the more classical role of oxygen and nutritional delivery.

Defining the cellular and microenvironmental cues that trigger EMT during cancer progression is important. Studies have shown increased expression of EMT markers at the tumor-stroma interface [15], [16] and stromal cells are now recognized as major players in cancer progression (reviewed in [17], [18]). The stromal compartment includes various cell types, e.g. fibroblasts (and myofibroblasts), immune cells and endothelial cells. Fibroblasts and myofibroblasts have received attention as important players in tissue morphogenesis and neoplasia [17], [46]. We have previously shown that breast cancer cells can generate non-malignant fibroblast-like cells that can facilitate growth and invasion of cancer cells [31]. Myofibroblast have been shown to induce EMT and tumor progression in a hepatocellular carcinoma mouse model through PDGF and TGF-beta signaling [47]. Recently, CD8 positive T cells have been shown to induce EMT in mouse mammary cancer cells. Following T cell-induced EMT, these cancer cells acquired cancer stem cell phenotype including increased CD44^high^/CD24^low^ ratio, drug resistance and increased tumorigenicity [48].

Although EMT can easily be recognized in monolayer culture of cells, recognizing these cells *in situ* is more troublesome, due to its transient nature. In contrast to monolayer cultures, 3D culture models capture more closely the *in vivo* situation [49]. Papers from our laboratory and others have shown the importance of 3D cultures to elucidate the functional role of the stroma as an instructive factor in normal breast morphogenesis and cancer progression [17], [18], [49], [50], [51]. Numerous cell lines, such as MCF10A and MCF-7, have been reported to be susceptible to EMT in traditional monolayer culture [52]. Our results, however, show that in 3D culture EMT induction by BRENCs is only achieved in selected cell lines, i.e. those harboring stem/progenitor characteristics (D492) and/or cell lines that have cancer initiating abilities (MDA-MB 231). We also show that primary metaplastic breast cancer cells, W2330 [31], can be facilitated to undergo EMT in 3D coculture with BRENCs. In contrast the luminal epithelial cell line D382, MCF10A and MCF-7 show no signs of EMT in coculture with BRENCs. Even though MCF10A has been shown to have a basal-like phenotype, they lack fundamental stem cell properties that D492 has, such as branching morphogenesis that may explain why they are non-responsive to endothelial induced EMT in 3D cultures.

Recent studies have shown that induction of EMT in immortalized human breast epithelial cells was associated with acquisition of cancer stem cell associated properties, measured by increased expression of CD44^high^/CD24^low^ cells accompanied by the ability to form mammosphere colonies in culture [6], [7]. In these studies, immortalized breast epithelial cells (HMECs) were induced to undergo EMT in 2D culture conditions with TGF-β1 or transfected with potent inducers of EMT such as snail, Twist or the ras oncogene. These studies are in line with our data where D492M show cancer stem cell and tumorigenic phenotype as evidenced by an increased ratio of CD44^high^/CD24^low^ cells, ability to form mammospheres, increased motility, anchorage independent growth and resistance against chemically induced apoptosis. It is noteworthy that in our study, D492, a cell line with epithelial stem cell properties, appear to lose the normal epithelial stem cell properties (i.e. generating differentiated luminal and myoepithelial cells and forming branching TDLU-like structures) after undergoing EMT and acquire a phenotype associated with cancer stem cells. This suggests an important difference between the properties of breast epithelial stem cells and epithelial cancer stem cells. Studies linking cancer stem cells and EMT also raise interesting questions about the cell renewal, developmental plasticity and signaling pathways involved in cancer progression.

In this paper we show that in basal like breast cancer, cells undergoing EMT are enriched in the vascular-rich areas and furthermore, we show that endothelial cells can directly induce EMT. This endothelial-induced EMT is at least partially facilitated by HGF making this a potential novel therapeutic target for patients with the basal-like subtype of breast cancer. Furthermore, our findings suggest a role for endothelial cells in basal-like breast cancer suggesting that therapy targeting the neovascular compartment might be relevant.

## Supporting Information

Figure S1 **Endothelial cells cultured in rBM appear as single, non proliferative but metabolically active cells.** Endothelial cells cultured for 10 days within rBM remain as single non proliferative but metabolically active as seen by the uptake of fluorescent labeled Ac-LDL (green). Insert shows single endothelial cells that have taken up Ac-LDL in higher magnification.
(TIF)

Figure S2 **Spindle-like colony formation increases proportionally with the amount of endothelial cells.** Increased number of BRENCs in coculture with D492 results in decreased and increased number of solid and spindle-like colonies. No effect was seen on branching colonies. AVG % of colonies +SEM in triplicate. *, p<0.05; **, p<0.01; compared to 5×10^4^ BRENCs.
(TIF)

Figure S3 **BRENCs facilitate mesenchymal phenotype in MDA-MB-231 a poorly differentiated breast cancer cell line.** To explore if BRENC could induce EMT in other cell types we set up cocultures of BRENCs (2×10^5^ cells) with MCF10A, MCF-7, D382 and MDA-MB-231 (500 cells). Coculture of BRENCs with MCF-10A, D382 and MCF-7 resulted in non-branching, non-EMT-like epithelial colonies. In contrast coculture of BRENCs with the highly malignant cancer cell line MDA-MB-231 resulted in large EMT-like colonies. Bar 100 µm.
(TIF)

Figure S4 **D492 and D492M share a common origin.** **A**, Origin of D492M confirmed by viral insertional analysis. D492 cell line contains a retroviral insertion of E6 and E7 genes. The insert site was identified (schematic) on chromosome 20q13.1 close to the gene PTP1N that codes for the protein tyrosine phosphatese 1B (PTP1B). PCR analyzes identified the same insert in D492M confirming its origin from D492. **B**, GFP positive D492 cells give rise to mesenchymal colonies in coculture with BRENCs. The origin of mesenchymal colonies from D492 was confirmed by using GFP positive D492. All colonies in the 3D culture were GFP positive. Bar = 100 µm.
(TIF)

Figure S5 **Gene expression analysis demonstrates global changes in D492-D492M transition.** Heat map showing the top 50 genes discriminating D492 and D492M. Red and green shows up- and down regulation of genes, respectively.
(TIF)

Figure S6 **Characterization of four mesenchymal-derived cell lines from D492.** D492-derived mesenchymal cell lines designed D492M1-M4 were characterized in terms of expression profile and for functional mesenchhymal properties. **A.** D492M1 show reduced expression of E-cadherin and EpCAM, weak expression of N-Cad and strong expression of fibronectin (FN) and vimentin. **B.** D492M-1 show increased migration compared to D492. **C.** Mesenchymal cell lines derived from D492 show advanced growth in soft agar. **D.** Summary of phenotypic and functional characteristics of D492M1-M4.
(TIF)

Figure S7 **BRENCs secreted HGF into the surrounding culture media.** BRENCs secreted HGF into the surrounding culture media as measured by ELISA. BRENCs secreted over four times higher concentration of HGF than D492 when cultured rBM.
(TIF)

Figure S8 **N-cadherin expression is prominent around vascular rich area of basal-like breast cancers.** Two basal like breast cancer were stained with antibodies against N-Cad and CD31. Figures show N-Cad high and N-Cad medium/low areas within the same cancer stained with N-Cad and CD31. Cells counterstained with heamotoxylin. Bar = 100 µm.
(TIF)

Methods S1 Supplementary material and methods.
(DOC)
