# Supplementary figures and images for: Endothelial Induced EMT in Breast Epithelial Cells with Stem Cell Properties
Source: PLoS One. 2011 Sep 6;6(9):e23833. doi: 10.1371/journal.pone.0023833 (PMC3167828; doi:10.1371/journal.pone.0023833)

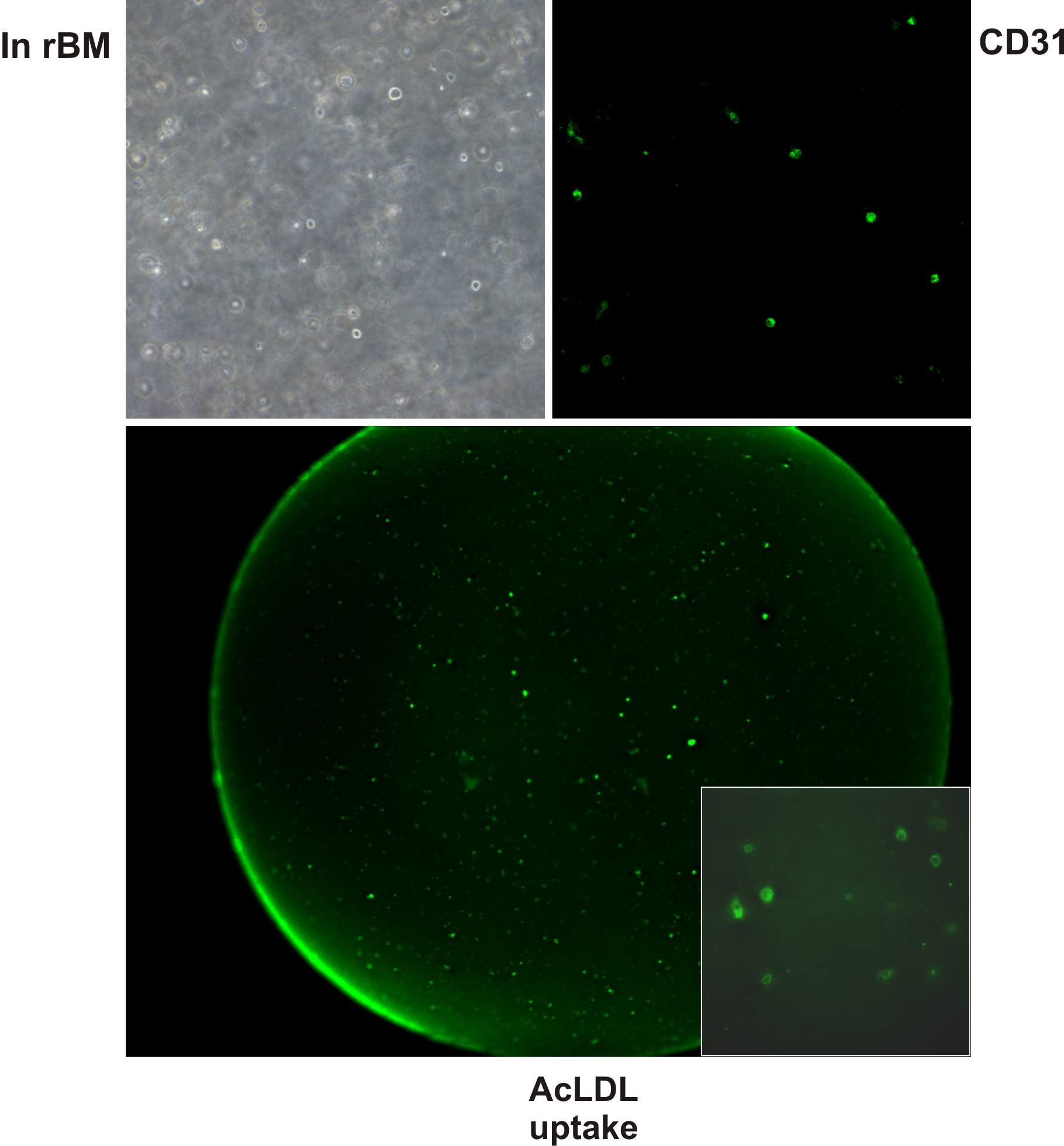

Supplement: Figure S1 — Endothelial cells cultured in rBM appear as single, non proliferative but metabolically active cells. Endothelial cells cultured for 10 days within rBM remain as single non proliferative but metabolically active as seen by the uptake of fluorescent labeled Ac-LDL (green). Insert shows single endothelial cells that have taken up Ac-LDL in higher magnification. (TIF) [file pone.0023833.s001.tif]

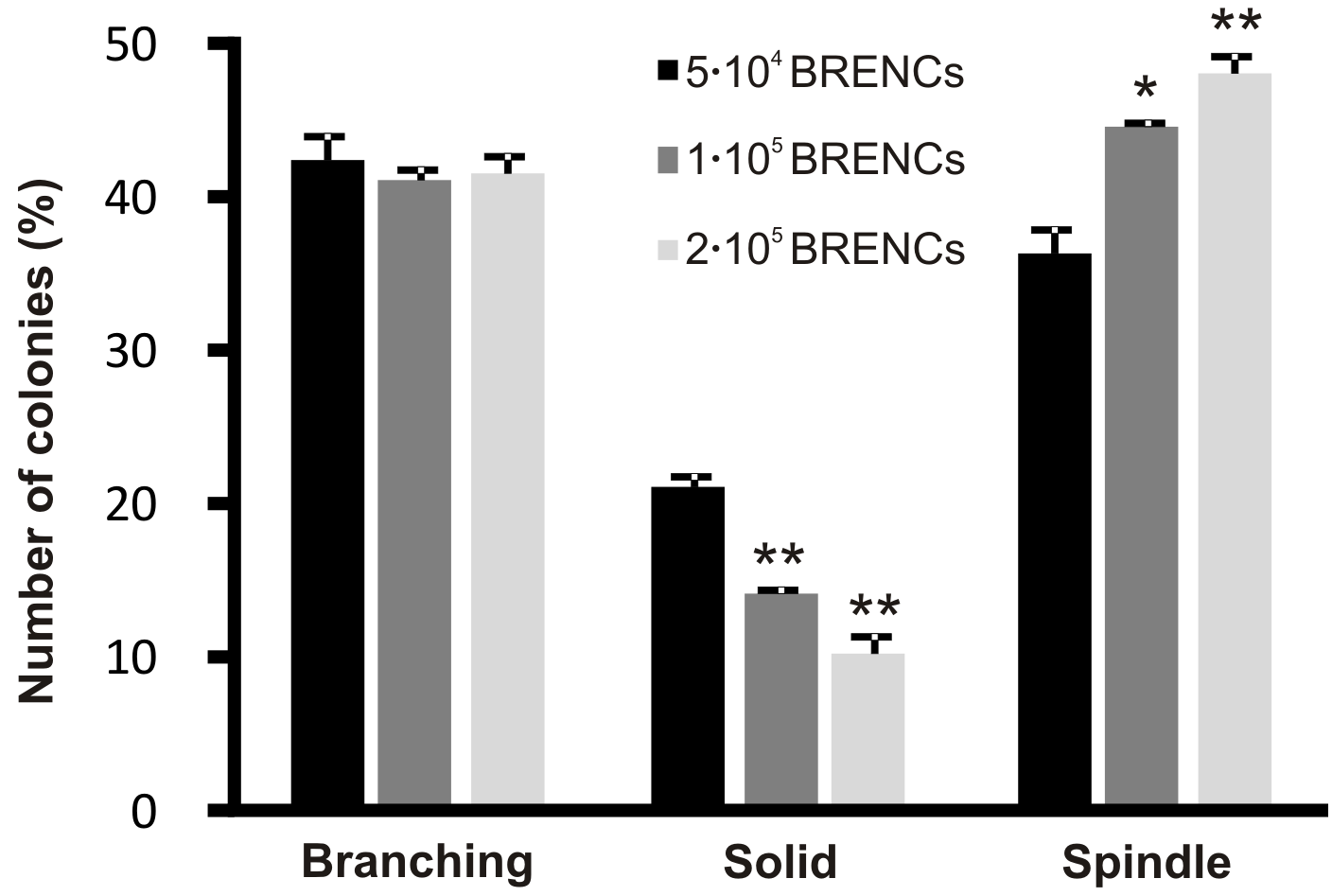

Supplement: Figure S2 — Spindle-like colony formation increases proportionally with the amount of endothelial cells. Increased number of BRENCs in coculture with D492 results in decreased and increased number of solid and spindle-like colonies. No effect was seen on branching colonies. AVG % of colonies +SEM in triplicate. *, p<0.05; **, p<0.01; compared to 5×10^4^ BRENCs. (TIF) [file pone.0023833.s002.tif]

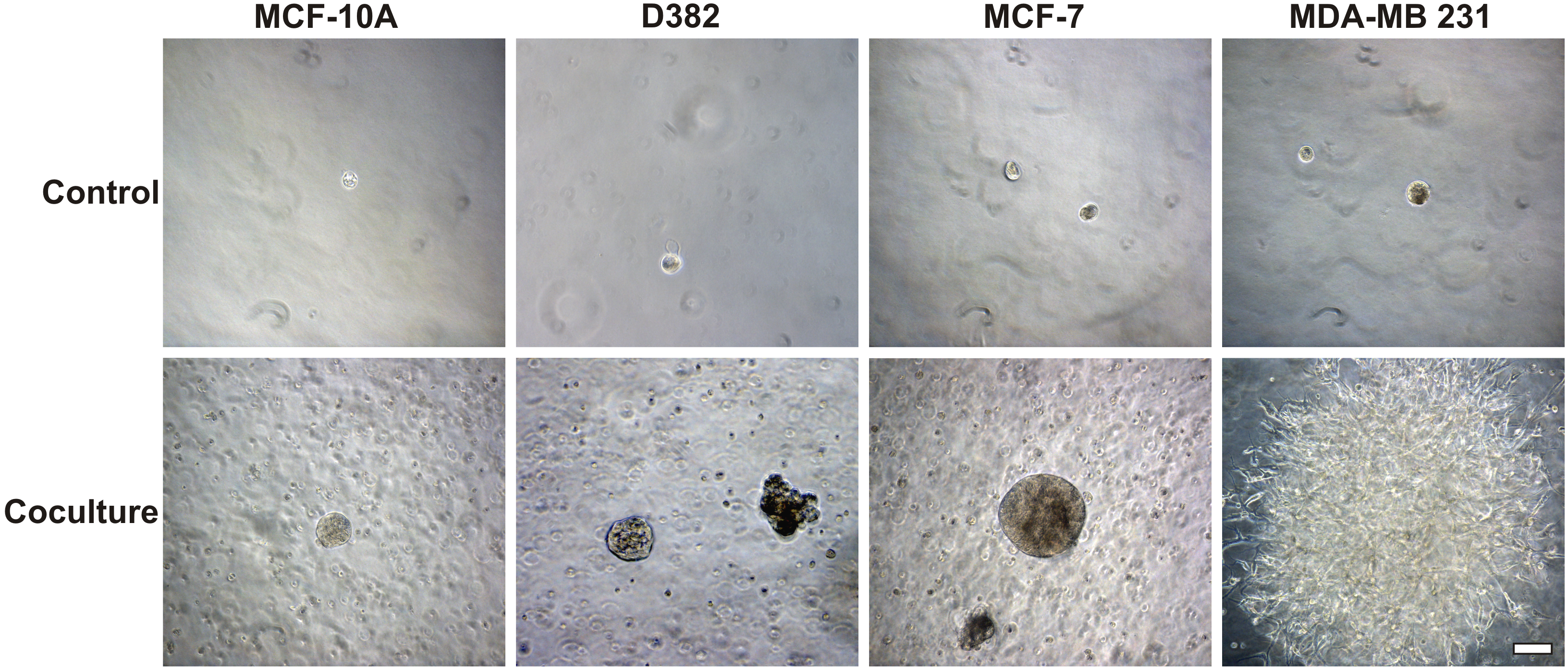

Supplement: Figure S3 — BRENCs facilitate mesenchymal phenotype in MDA-MB-231 a poorly differentiated breast cancer cell line. To explore if BRENC could induce EMT in other cell types we set up cocultures of BRENCs (2×10^5^ cells) with MCF10A, MCF-7, D382 and MDA-MB-231 (500 cells). Coculture of BRENCs with MCF-10A, D382 and MCF-7 resulted in non-branching, non-EMT-like epithelial colonies. In contrast coculture of BRENCs with the highly malignant cancer cell line MDA-MB-231 resulted in large EMT-like colonies. Bar 100 µm. (TIF) [file pone.0023833.s003.tif]

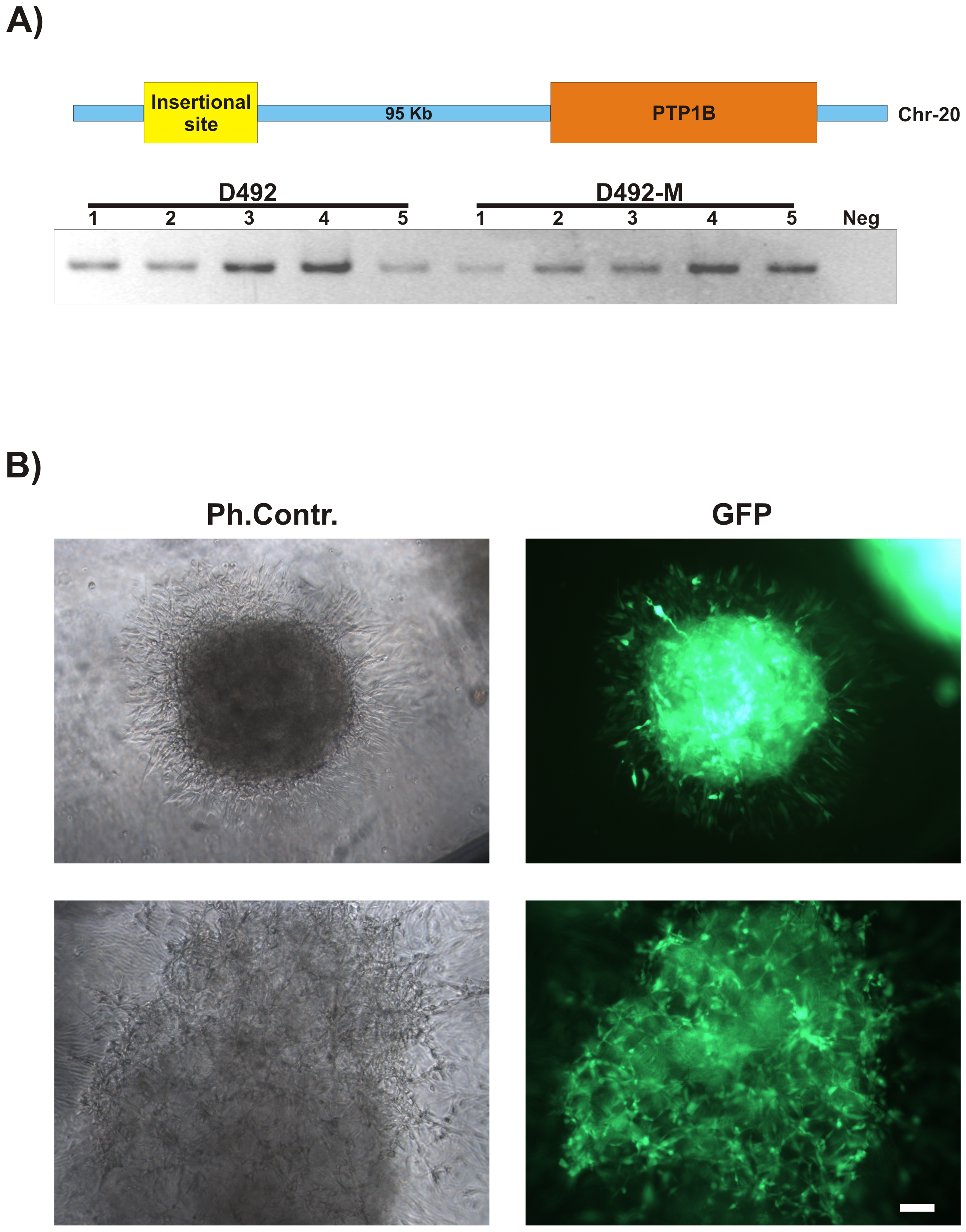

Supplement: Figure S4 — D492 and D492M share a common origin. A, Origin of D492M confirmed by viral insertional analysis. D492 cell line contains a retroviral insertion of E6 and E7 genes. The insert site was identified (schematic) on chromosome 20q13.1 close to the gene PTP1N that codes for the protein tyrosine phosphatese 1B (PTP1B). PCR analyzes identified the same insert in D492M confirming its origin from D492. B, GFP positive D492 cells give rise to mesenchymal colonies in coculture with BRENCs. The origin of mesenchymal colonies from D492 was confirmed by using GFP positive D492. All colonies in the 3D culture were GFP positive. Bar = 100 µm. (TIF) [file pone.0023833.s004.tif]

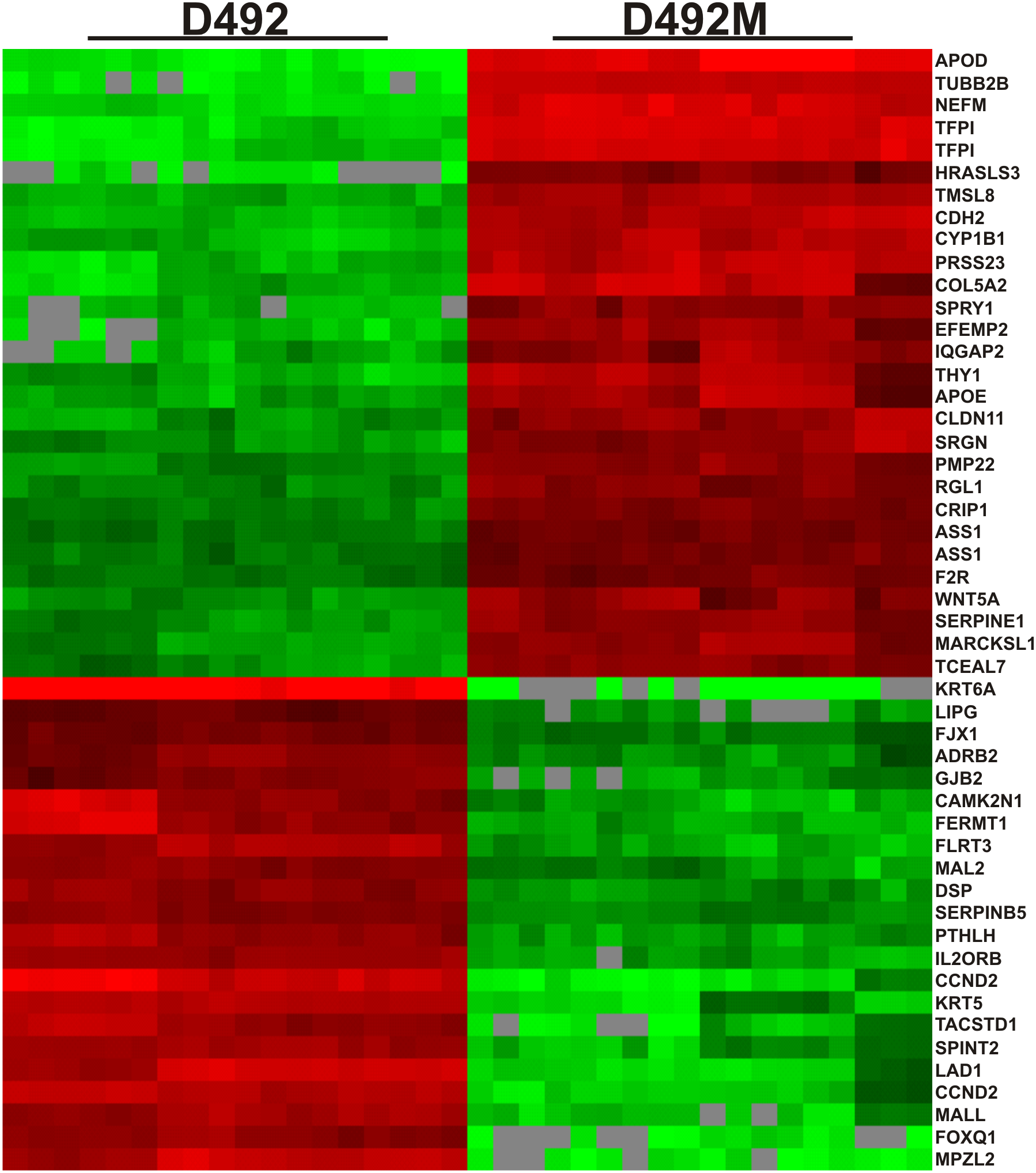

Supplement: Figure S5 — Gene expression analysis demonstrates global changes in D492-D492M transition. Heat map showing the top 50 genes discriminating D492 and D492M. Red and green shows up- and down regulation of genes, respectively. (TIF) [file pone.0023833.s005.tif]

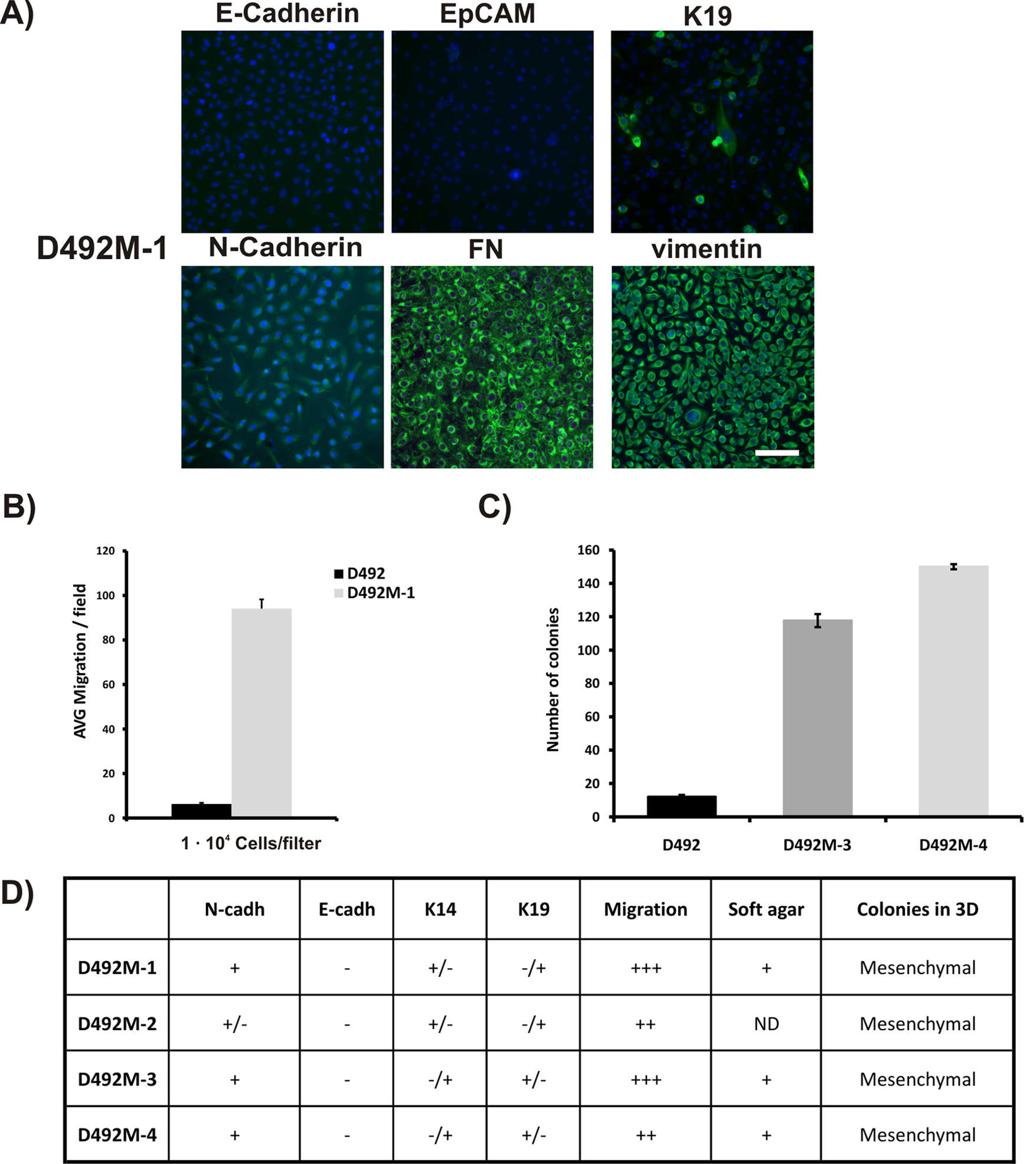

Supplement: Figure S6 — Characterization of four mesenchymal-derived cell lines from D492. D492-derived mesenchymal cell lines designed D492M1-M4 were characterized in terms of expression profile and for functional mesenchhymal properties. A. D492M1 show reduced expression of E-cadherin and EpCAM, weak expression of N-Cad and strong expression of fibronectin (FN) and vimentin. B. D492M-1 show increased migration compared to D492. C. Mesenchymal cell lines derived from D492 show advanced growth in soft agar. D. Summary of phenotypic and functional characteristics of D492M1-M4. (TIF) [file pone.0023833.s006.tif]

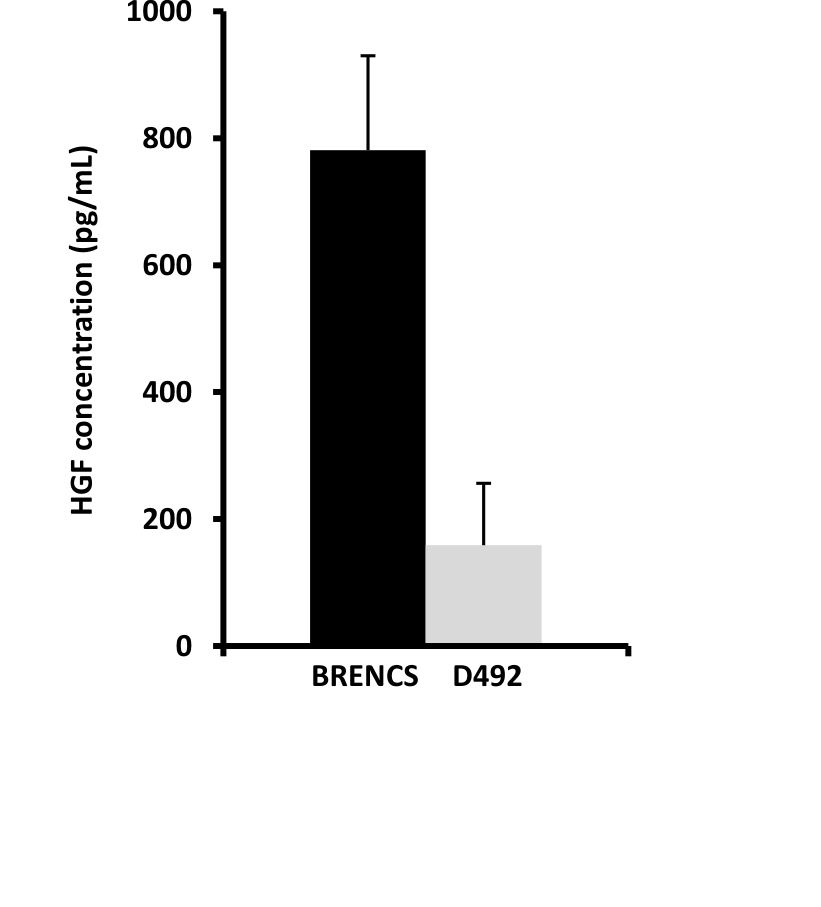

Supplement: Figure S7 — BRENCs secreted HGF into the surrounding culture media. BRENCs secreted HGF into the surrounding culture media as measured by ELISA. BRENCs secreted over four times higher concentration of HGF than D492 when cultured rBM. (TIF) [file pone.0023833.s007.tif]

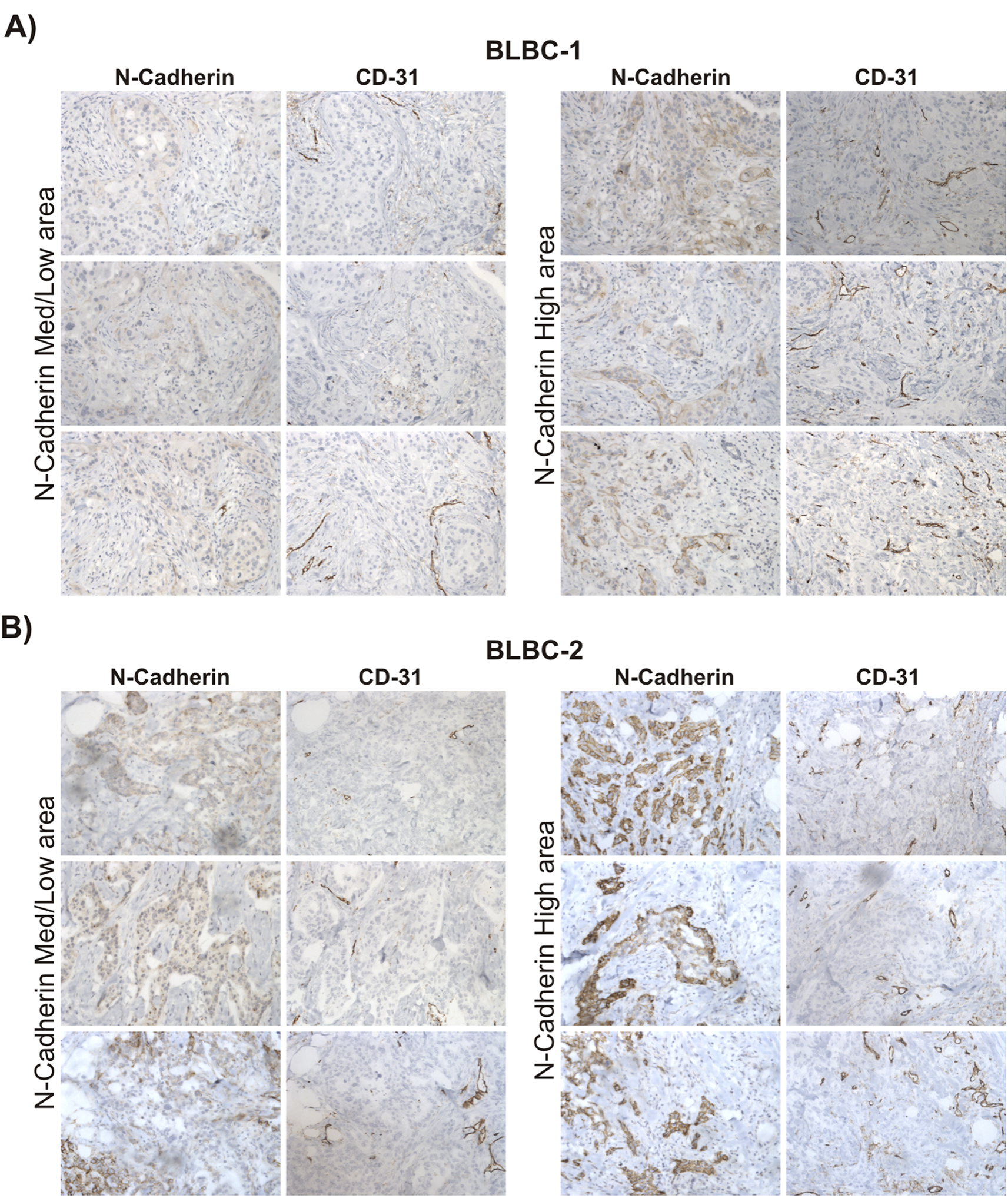

Supplement: Figure S8 — N-cadherin expression is prominent around vascular rich area of basal-like breast cancers. Two basal like breast cancer were stained with antibodies against N-Cad and CD31. Figures show N-Cad high and N-Cad medium/low areas within the same cancer stained with N-Cad and CD31. Cells counterstained with heamotoxylin. Bar = 100 µm. (TIF) [file pone.0023833.s008.tif]
