# Supplementary material for: Endothelial Induced EMT in Breast Epithelial Cells with Stem Cell Properties
Source: PLoS One. 2011 Sep 6;6(9):e23833. doi: 10.1371/journal.pone.0023833 (PMC3167828; doi:10.1371/journal.pone.0023833)
Supplement: Methods S1 — Supplementary material and methods. (DOC) [file pone.0023833.s009.doc]

**Supplementary material and methods**

**Cell culture**

The breast epithelial stem cell line D492 and daughter cell line D492M were maintained in H14 medium (Briand, Petersen et al. 1987), consisting of DMEM/F12, 50 IU/ml penicillin, 50 µg/ml streptomycin (Invitrogen), 250 ng/ml insulin, 10 µg/ml transferrin, 2,6 ng/ml sodium selenite, 0,1 nM estradiol, 0,5 µg/ml hydrocortisone, 5 µg/ml prolactin (SIGMA) and 10 ng/ml EGF (Peprotech). Luminal epithelial cell line referred to as D382 and MCF10A were also maintained on H14 medium. MDA-MB 231 was cultured on RPMI-1640 supplemented with 5% FBS. W2320 and MCF-7 on DMEM/F12 with 5% FBS. The MCF-7, MCF10A and MDA-MB-231 cell lines where purchased from ATCC and are routinely authenticated with genotype profiling according to ATCC guidelines. To further ensure cell line integrity D492, D492M and D382 cell lines were analyzed with the same method.

**Generation of GFP positive D492 cells**

Lentiviral pGIPZ vector (RHS4346) expressing green fluorescent protein (GFP) (Open Biosystems, Huntsville, AL) was transfected into HEK-293T cells using Arrest-In transfection reagent (ATR1740; Open Biosystems) according to instructions. Virus-containing supernatants were collected at 48 hours after transfection and target cells infected in the presence of 8ug/ul polybrene. 24 hours later, drug selection was done with 3ug/ul puromycin to establish stable cell line expressing GFP.

**Preparation of 3D cocultures**

Coculture experiments were carried out in 24 well culture plates (BD Falcon) with 500 D492 cells alone (monoculture) and with , 5x10^4^, 1x10^4^ and 2x10^5^ BRENCs (cocultures). The two cell types were mixed and suspended in 300µl rBM and cultured in EGM5 for 15 days. Cocultures of BRENCs with normal breast epithelial lines MCF10A and D382, estrogen receptor positive breast cancer cell line MCF-7, basal-like / EMT breast cancer cell line MDA-MB-231 and primary metaplastic breast cancer cell line W2320 were done with 500 epithelial cells and 2x10^5^ BRENCs in EGM5 medium.

**Isolation of 3D coculture colonies, replating and secondary 3D coculture**

Branching, solid and spindle-like structures were isolated from 3D cocultures with gentle shaking on ice in PBS - EDTA (5mM) solution. Single structures were placed in a 24 well plate and cultured on H14 medium. Monolayer cultured cells from branching, solid and spindle like colonies were then put back into 3D coculture of 500 cells with 5x10^4^ BRENCs in rBM.

**ELISA and additional blocking experiments**

BRENCS and D492 were seeded in 3D monocultures in rBM and the HGF concentration in the culture media was determined using comercially available HGF ELISA kit (DHG00; R and D, MN, USA) according to instructions. ALK5 kinase inhibitor (SB431542, Tocris Bioscience) was used to block signals through the ALK5 receptor and was diluted in the rBM (10µM) and in the medium (10µM) in coculture of 500 D492 cells and 5x10^4^ BRENCs. We also blocked TGFβ1(8µg/ml) in 3D coculture with a neutralizing antibody (ab10517, Abcam) in the rBM and in the medium.

**Microvessel density scoring**

Microvessel counting was conducted as previously described [1,2]. Briefly, microvessel density was evaluated by immunohistochemistry of tumor vessels for CD31 in whole tissue sections. An immunopositive cell or cluster of cells clearly separated from adjacent clusters, was considered an individual vessel. Microvessels were counted in three different areas of low and high N-cadherin expression, respectively, in three different biopsies in a 200x field.

**Endothelial uptake of AcLDL in 3D rBM cultures**

Endothelial cells have the ability to take up AcLDL and this trait has become routine to identify them in culture. BRENCs were treated with 15 µg/ml AcLDL conjugated to A488 fluorecent dye (Invitrogen) for 4 hrs. The uptake of AcLDL-A488 was monitored on day 10 in 3D cultures of BRENCs.

**Retroviral insertion analysis**

The D492 cell line was initially established by transfection with a retroviral vector containing the E6 and E7 oncogenes and the neomycin resistant gene for selection [3]. To identify the genomic insertion site of the E6/E7 containing retrovirus we performed an inverse PCR (I-PCR) (Suzuki et al., 2002) by using 5 μg of cell line DNA digested with 60 U of BamHI overnight in 40 μl. After heat inactivation DNA was diluted to 200 μl, circularized by ligation with T4 DNA ligase at 16°C overnight, ethanol precipitated, and resuspended in 30 μl of Tris-EDTA. PCR was performed in 25 μl with 1 μl of the DNA template, 0,2 mM deoxynucleoside triphosphates, 10 pmol of each primer, 1,3 U of Expand Long Template Polymerase, and Expand Buffer 1 (Roche). The primers used were I-1F (CTAGCTTGCCACCTACGGGT) and I-1R (TGAGGAAATTGAGGCACAGC). The cycling conditions were 94°C for 2 min, followed by 10 cycles of 94°C for 10 s, 65°C for 30 s, and 68°C for 6 min and 20 cycles of 94°C for 10 s, 65°C for 30 s, and 68°C for 6 min with a 20-s autoextension and a final extension at 68°C for 10 min. Amplified products were cloned into the TA cloning TOPO vector (Invitrogen) and clones selected and sequenced. We identified with this method a single insertion on chromosome 20, 95 kb upstream of the gene PTPN1 (encoding for the phosphatase PTP1B).

**Gene expression analysis**

RNA was isolated from D492 and D492M at 50% and 90% confluency in monolayer culture using RNeasy mini kit (QIAGEN). Experiments were conducted in triplicate, on three different time points (36 samples). RNA was analysed on NanoDrop ND-1000 spectrophotometer and run on Agilent 2100 Bioanalyzer chip. Microarray analysis was carried out using the Illumina BeadChip expression microarray (HumanWG-6 v3.0) platform. The data was background subtracted and normalized using cubic spline with all samples as a group using BeadStudio. Probes were quality filtered such that if p detect >0,01 then the intensity was replaced with a missing value. Probes with missing values for all 36 hybridizations were omitted from future analysis. This left 16547 probes which had p value <=0,01 in at least one hybridization. To identify differentially expressed genes we used the MeV software (www.tm4.org) and the significance of microarrays (SAM) method [4]. Genes that had detectable expression levels in 50% of the samples were used in the comparison. All raw data are available at [http://stofnanir.hi.is/rle/sites/stofnanir.hi.is.rle/files/EMT-expression%20](http://stofnanir.hi.is/rle/sites/stofnanir.hi.is.rle/files/EMT-expression )profile%20D492-vs-D492M.xlsx

**Supplementary references**

1. Lopes N, Sousa B, Vieira D, Milanezi F, Schmitt F (2009) Vessel density assessed by endoglin expression in breast carcinomas with different expression profiles. Histopathology 55: 594-599.

2. Marinho A, Soares R, Ferro J, Lacerda M, Schmitt FC (1997) Angiogenesis in breast cancer is related to age but not to other prognostic parameters. Pathol Res Pract 193: 267-273.

3. Gudjonsson T, Villadsen R, Nielsen HL, Ronnov-Jessen L, Bissell MJ, et al. (2002) Isolation, immortalization, and characterization of a human breast epithelial cell line with stem cell properties. Genes Dev 16: 693-706.

4. Tusher VG, Tibshirani R, Chu G (2001) Significance analysis of microarrays applied to the ionizing radiation response. Proc Natl Acad Sci U S A 98: 5116-5121.
